# Sporadic inclusion body myositis: the genetic contributions to the pathogenesis

**DOI:** 10.1186/1750-1172-9-88

**Published:** 2014-06-19

**Authors:** Qiang Gang, Conceição Bettencourt, Pedro Machado, Michael G Hanna, Henry Houlden

**Affiliations:** 1 Department of Molecular Neuroscience, Institute of Neurology, University College London, Queen Square, London WC1N 3BG, UK; 2 MRC Centre for Neuromuscular Diseases, Institute of Neurology, University College London, London WC1N 3BG, UK

**Keywords:** Inclusion body myositis, Inclusion body myopathy, IBM, Genes, Genetics, Exome sequencing

## Abstract

Sporadic inclusion body myositis (sIBM) is the commonest idiopathic inflammatory muscle disease in people over 50 years old. It is characterized by slowly progressive muscle weakness and atrophy, with typical pathological changes of inflammation, degeneration and mitochondrial abnormality in affected muscle fibres. The cause(s) of sIBM are still unknown, but are considered complex, with the contribution of multiple factors such as environmental triggers, ageing and genetic susceptibility. This review summarizes the current understanding of the genetic contributions to sIBM and provides some insights for future research in this mysterious disease with the advantage of the rapid development of advanced genetic technology. An international sIBM genetic study is ongoing and whole-exome sequencing will be applied in a large cohort of sIBM patients with the aim of unravelling important genetic risk factors for sIBM.

## Introduction

Sporadic inclusion body myositis (sIBM) is the commonest form of idiopathic inflammatory myopathy among individuals aged over 50 [1]. It has a male predominance and a prevalence of 1–71 people per million inhabitants has been reported in different populations, rising up to 139 per million among people over 50 years old (Table 1) [1-10]. These figures may be substantially underestimated due to frequent initial misdiagnosis of this disease [11-13]. Clinically, sIBM is characterised by progressive quadriceps femoris and deep finger flexors weakness and atrophy [11,12]. Many patients become wheelchair dependent and severely disabled 10–15 years after symptom onset, although disease progression is heterogeneous [12,13]. Dysphagia, due to oesophageal and pharyngeal muscle involvement, affecting up to 80% of sIBM patients, can be a significant problem predisposing to malnutrition, pulmonary aspiration and pneumonia [11-13]. Muscle tissue in sIBM is characterised by the presence of inflammatory and degenerative features. Inflammatory features include endomysial inflammation, partial invasion (invasion of non-necrotic fibres by inflammatory cells) and up-regulation of major histocompatibility complex (MHC) class I [14]. Degenerative features include the formation of rimmed vacuoles, tubulofilaments seen on electron microscopy and the accumulation of many myotoxic proteins (including amyloid, p62 and TAR DNA-binding protein-43 (TDP-43)), termed as ’inclusions’ [15]. The observation of ragged-red, cytochrome c oxidase (COX) deficient and succinate dehydrogenase positive fibres reflects mitochondrial changes in sIBM muscle tissue [16].

**Table 1:** Prevalence of sporadic IBM in different populations

**Study**	**Region**	**Diagnostic criteria**	**Prevalence (per million population)**	**Prevalence > 50 yrs (per million population)**
Lingberg et al. [2]	Sweden	Biopsy and clinical data	2.2	n/a
Kaipiainen-Seppanen and Aho [4]	Finland	Not mentioned	0.9	n/a
Philips et al. [5]	Australia	Griggs et al. criteria	9.3	35.3
Badrising et al. [3]	Netherlands	ENMC criteria	4.9	16
Felice and North (Connecticut, USA) [6]	Connecticut, USA	Griggs et al. criteria	10.7	28.9 (>45 yrs)
Needham et al. [1]	Western Australia	Clinical and biopsy criteria	14.9	51.2
Wilson et al. [7]	Olmsted County, USA	Griggs et al. criteria	71	n/a
Oflazer et al. [8]	Istanbul, Turkey	Own criteria from the study (biopsy and clinical data)	1.0	6.0
Suzuki et al. [9]	Japan	Clinical and biopsy criteria	1.3 (in 1991)	n/a
			9.8 (in 2003)	n/a
Tan et al. [10]	South Australia	Biopsy and clinical data	50.5	139.3

The clinical diagnosis of sIBM is confirmed by muscle biopsy assisted by the clinical phenotype, electromyography (EMG), determination of serum muscle-enzyme levels and muscle imaging with MRI [11]. Currently, the Griggs Criteria [17,18], the European Neuromuscular Centre (ENMC) Criteria [19,20] and the MRC Centre for Neuromuscular Diseases Criteria [21,22] are the most widely used diagnostic criteria for sIBM. However, the aetiology of sIBM is still unclear and no effective treatment is available [11,23].

sIBM is a complex disease. The primary pathogenic mechanism is still hotly debated and it is believed that many factors including environmental factors contribute to the disease process. There are many clinically and/or histologically sIBM-like muscle diseases with known genetic defects, which may provide clues to understand the pathogenesis of sIBM. In this review we will comprehensively summarize known and candidate genetic susceptibility risk factors for sIBM.

## Muscle disorders similar to sIBM

### Familial inclusion body myositis

There are several IBM case reports of two or more affected siblings in the same family [24-26] and one report of affected twins [27]. Because of the clinical and histological similarities with sIBM these cases have been called familial inclusion body myositis (fIBM)*.* Human leukocyte antigen (*HLA*) class II genotypes were reported in a strong association with some families, particularly the *DR3* allele (DRB*0301/0302) in four western families [24,25] and the *DR15(2)*/*DR4* (DRB1*1502/0405) in two Japanese sisters [26]. The fIBM and sIBM share not only similar clinical, biological, MRI and histopathological features but also similar genetic markers, which indicate the possibility that the two subsets of IBM may share the same susceptibility determinants to the disease development and also highlight the genetic predisposition for sIBM pathogenesis.

### Hereditary inclusion body myopathies

Hereditary inclusion body myopathies (hIBM) include several autosomal-recessive and autosomal-dominant muscle disorders with various clinical presentations, but with a number of pathological features similar to those of sIBM, including rimmed vacuoles, protein accumulations and tubulofilaments on electron microscopy. Patients with hIBM can be distinguished from sIBM by the earlier age of onset, rarity of inflammatory features and negative MHC class I expression, hence the term ‘myopathy’ instead of ‘myositis’ [28]. The hIBM can be grouped according to their mode of inheritance and genetic mutations (see hIBM subtypes in Table 2, and details in a previous IBM genetics review [29]).

**Table 2:** **Hereditary inclusion body myopathy subtypes (further detailed by Needham et al.**[29]**)**

**Hereditary IBM (hIBM) subtypes**	**OMIM#**	**Disease inheritance**	**Genes**	**Function of coding proteins**
Inclusion body myopathy 2 - hIBM2 (distal myopathy with rimmed vacuoles -DMRV/Nonaka myopathy)	600737 (605820)	Autosomal-recessive	UDP-N-acetylgucosamine-2-epimerase/N-acetylmannosamine kinase (*GNE*) gene	A rate-limiting enzyme in the sialic acid biosynthetic pathway, involved in sialylation of muscle glycoproteins, and cellular homeostasis
A leukoencephalopathy and a vacuolar myopathy resembling IBM	n/a	Autosomal-recessive	Laminin alpha 2 *(LAMA2)* gene [30]	An extracellular protein of basement membrane, mediates the attachment, migration, and organization of cells into tissues during embryonic development
hIBM with congenital joint contractures, ophthalmoplegia and rimmed vacuoles – hIBM3	605637	Autosomal-dominant	Myosin heavy chain IIa (*MYH2;* previously known as *MHCIIa*) gene	A member of Class II or conventional myosin heavy chains, functions in skeletal muscle contraction
Inclusion body myopathy with early-onset Paget’s disease of the bone (PDB) and frontotemporal dementia (FTD) (IBMPFD)	167320	Autosomal-dominant	Valosin-containing protein (*VCP*) gene	A member of the ‘ATPases associated with a variety of activities (AAA-ATPase)’ superfamily, is involved in a variety of cellular activities, such as cell cycle control, membrane fusion and the ubiquitin-proteasome degradation pathway

### Other rimmed vacuolar myopathies

Beyond the above conditions, there are various other neuromuscular disorders showing rimmed vacuoles in the muscle tissue, which make the differential diagnosis of sIBM even wider. Mutated genes have also been found in these called ‘rimmed vacuolar myopathies’, such as oculopharyngeal muscular dystrophy (OMIM#164300), X-linked recessive Emery-Dreifuss muscular dystrophy (OMIM#310300), limb girdle muscular dystrophy (LGMD) type 2G (OMIM#601954), LGMD type 1A (OMIM#159000), LGMD type 1G (OMIM#609115), and rigid spine syndrome (OMIM#602771) [29]. Some intracellular Alzheimer’s-like protein accumulations, including amyloid proteins, amyloid-β Protein Precursor (AβPP), phosphorylated tau and presenilin-1, among others, have been found in extensive overlap between sIBM and myofibrillar myopathies [31].

Overall, these common features raise the possibility that sIBM and these diseases share certain etiologic factors. Indeed, a recent study [32], reported that the p.V805A variant in *MYH2* (gene associated with hIBM3) significantly increased the risk of developing sIBM (RR = 12.2). However, none of the patients with that variant reported joint contractures or external ophthalmoplegia, a family history of IBM or consanguineous marriage [32]. This was the first report of a *MYH2* gene mutation described in non-familial cases. Immunohistochemical studies of myosin heavy chain isoforms showed atrophy or loss of muscle fibres expressing myosin heavy chain IIa or IIx, which may provide important clues for establishing the pathogenicity of this variant. In the same study, a novel missense mutation c.1719G > A (p.V566M) in the LIM domain binding 3 gene (*LDB3*, also known as *ZASP*; causative gene for zaspopathy, a subtype of myofibrillar myopathies), coding for Z-band alternatively spliced PDZ-motif-containing protein, was reported in one of the patients [32]. This finding indicates *LDB3* mutations might cause abnormalities in the Z-band in skeletal muscle resulting in the disease observed in this patient. Further studies with a larger number of cases will be needed to clarify the contribution of these genes to the pathogenesis of sIBM. In addition, investigations should be carried out on more hereditary myopathies candidate genes, including α-B crystalline (*CRYAB*), dystrophin (*DMD*) and myotilin (*MYOT*) in the future.

## Pathogenesis of sIBM and its predisposing genetic factors

**Figure 1:**
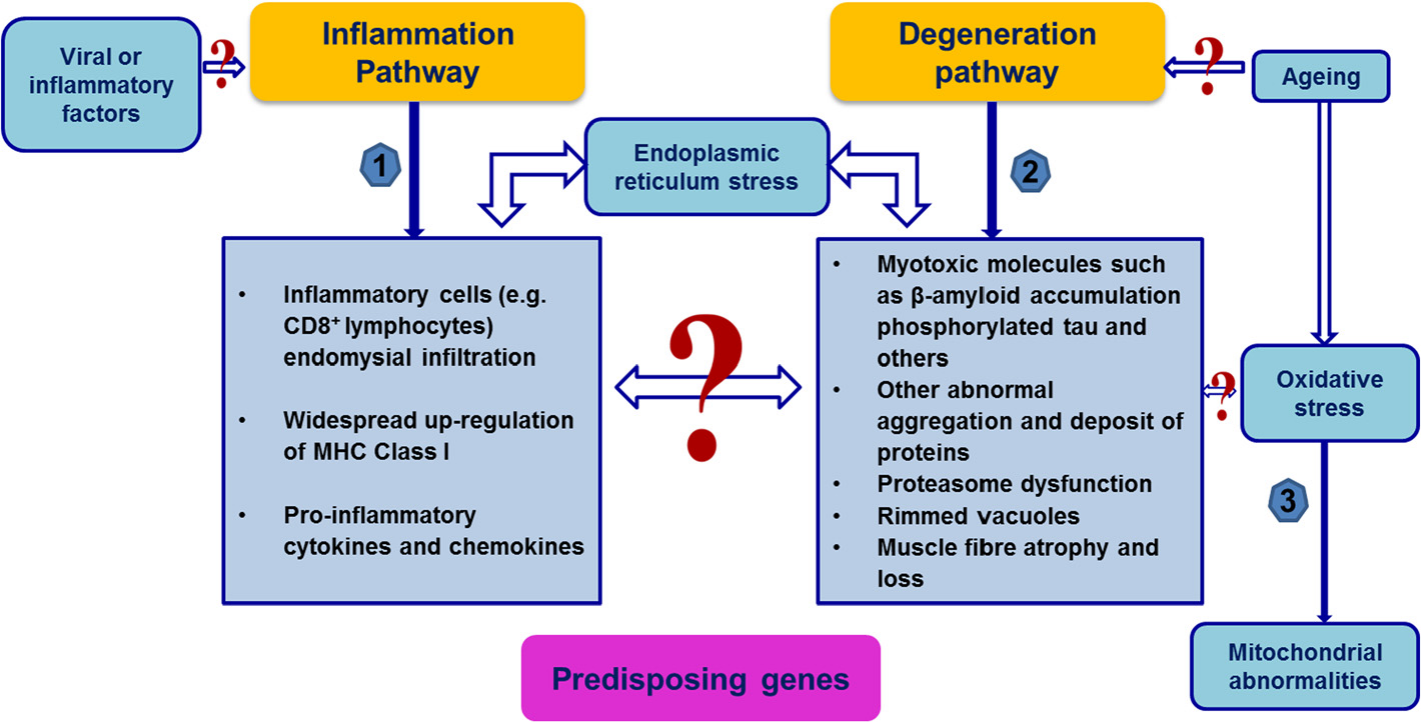
**Possible pathogenic pathways of sIBM – multifactorial mechanisms involved.** An inflammation pathway and a degeneration pathway constitute the two most popular theories for the pathogenesis of sIBM. Ageing is also considered an important factor contributing to mitochondrial dysfunction in result of oxidative stress in the muscle tissue. However, it is still not clear which is the primary cause of the disease, and how these potential pathways interact. Predisposing genes could also contribute to the development and progression of the disease.

Currently there are two most popular theories for the pathogenesis of sIBM – an autoimmune pathway and a degeneration pathway. In addition, ageing is also considered an important factor contributing to mitochondrial abnormalities (Figure 1).

The theory of an inflammation pathway stands for viral or inflammatory triggering factors leading to the clonal expansion of CD8^+^ T cells and T cell-mediated cytotoxicity, which ultimately result in damage or death of muscle fibres [33]. This theory is supported by the increased occurrence of sIBM in the presence of autoimmune disorders and HIV and HTLV-1 infection [34]. Degenerative changes and endoplasmic reticulum (ER) stress are considered as secondary mechanisms induced by increased intracellular cytokines and chemokines [33]. The degree of mitochondrial changes and muscle atrophy were suggested to be strongly correlated with the severity of inflammation in a recent study [35].

However, sIBM is poorly responsive to even vigorous immunosuppression. Even where there is histopathological evidence that inflammation was reduced, this was not accompanied by clinical improvement. Therefore, some investigators are supporting a degenerative hypothesis over inflammation as the primary pathogenesis of sIBM. The identification of aberrant protein aggregates in sIBM vacuolated muscle fibres has shown the remarkable parallels of those features in brain tissue of Alzheimer’s disease (AD) and Parkinson’s disease with Lewy bodies. This theory suggests that inflammation is secondary to the degeneration-associated processes in sIBM muscle fibres [36] including: 1) multiple protein aggregates [37], 2) abnormal accumulation of lipoprotein receptors and free cholesterol [38], 3) oxidative stress [39], 4) inhibition of the ‘ubiquitin-proteasome system’ (UPS) [40], 5) endoplasmic reticulum stress [41], and 6) impaired autophagy-lysosome pathway [41]. Furthermore, myonuclear disintegration is also involved in the pathogenic process leading to the formation of rimmed vacuoles. This results in a severe consequence that is a progressive reduction of the number of myonuclei and further progressive muscle atrophy [42].

Despite sIBM not being an inherited Mendelian disease, multiple genetic risk factors are being shown likely to play important roles in the development and progression of sIBM. A list of possible susceptibility genes that could be important candidate genes for understanding the pathogenesis of sIBM is shown in Table 3. Furthermore, the prevalence of sIBM differs between different ethnic populations. This is likely due to differences in genetic makeup of different racial/ethnic groups and differences in the environmental factors of different geographical regions [29].

**Table 3:** Summary of possible susceptibility genes for sIBM based on current research and discussed in this review

**Classification**	**Regions/Genes**
Immune-associated genes	MHC region [43]; *NT5C1A* gene [44].
Degenerative-associated genes	*APP* gene [45]; *PSEN* gene [46]; *DYSF* gene [47]; *APOE* gene [48]; *MAPT* gene [49]; *PRNP* gene [50]; *SERPINA3* gene [51]; *TARDBP* gene [52]; *hnRNPA1* and *hnRNPA2B1* genes [53]; *C9orf72*[54]*.*
mtDNA-associated sequences/genes	mtDNA deletions [55]; Nuclear coding mitochondrial genes: *TYMP*, *SLC25A4*, *C10orf2*, *POLG1,* and *TOMM40* gene [16,56].

### Genetic factors related to inflammatory changes in sIBM

#### Major histocompatibility complex

Garlepp and colleagues first reported the strong association of *HLA-DR3* and the extended 8.1 ancestral haplotype (*AH*) with sIBM [57]. This association has subsequently been confirmed in a series of further studies in Caucasians with HLA-A*01, -B*0801, -DRB1*0301, -DRB1*0101, -DRB1*1301, -DQB1*0201, -DQA1*05[58-65]. Furthermore, Rojana-udomsart and colleagues reported that the risk factors HLA-DRB1*0301 and *1301 also influence on the age at onset and the severity of muscle weakness [65]. These results indicate that the interactions at *HLA-DRB1* locus may contribute to both disease susceptibility and clinical phenotype. Other alleles and haplotypes have also been associated with increased risk of sIBM, including the 35.2AH (HLA-B*3501, -DRB1*0101) in Caucasians [63] and the 52.1AH (HLA-B*5201, DRB1*1502) in Japanese [66]. Interestingly, a number of HLA alleles and haplotypes, including *HLA-DR53* and HLA-DRB1*0401, *0701, *0901, *1101, *1501, HLA-DRB1*0301/*0401 and HLA-DRB1*0301/*0701 diplotypes, and HLA-DRB1*1501, were found protective for sIBM [58,65,67].

A potential region of MHC-associated susceptibility genes was first defined between *HLA-DR* and *C4*[43], and then it was suggested in the border of the Class II and III regions, between *HLA-DRB1* and pre-B-cell leukemia homeobox2 (*PBX2*, also known as *HOX12*) [63]. A further recombination mapping was applied consisting of three separate cohorts to refine the probably 8.1 AH-derived sIBM susceptibility region from butyrophilin-like MHC class II-associated gene (*BTNL2*) to telomeric of *HLA-DRB1* including three protein-coding genes – part of *BTNL2, HLA-DRA* and *HLA-DRB3*[68]. Other genes in this region have also been considered as candidate genes, such as notch 4 gene (*NOTCH4*; a transmembrane receptor which regulates cell fate decisions), advanced glycosylation end product-specific receptor gene (*AGER*, previously known as *RAGE*), testis-specific basic protein gene (*C6orf10*, also known as *TSBP*), and G-protein signalling modulator 3 gene (*GPSM3*, previously known as *G18*) [43,63].

Two *NOTCH4* gene polymorphisms (rs422951 and rs72555375) have shown a strong association (OR > 2) with sIBM in two independent Caucasian cohorts [69]. These variants (or other variants in linkage disequilibrium with them) may play a role in sIBM pathogenesis by generating antigenic peptides and can be regarded as markers for sIBM susceptibility. They may also influence the binding and affinity of notch 4 receptor and the presentation of the gene product at the cell surface, respectively [69].

The MHC contains important genetic contents which have been reported as being associated with sIBM and among them 8.1AH which is predominant in Caucasians, supporting an immune-mediated mechanism underlying sIBM. Identification of the actual genes within the MHC will be crucial to understand the role of MHC in the pathogenesis of sIBM.

#### Other autoimmune factors

An autoantibody against the cytoplasmic 5′-nucleotidase 1A (cN1A; *NT5C1A*) was recently identified in sIBM by two independent groups [44,70,71]. cN1A, as a member of the family of 5′-nucleotidases, is a muscle-specific enzyme catalysing the hydrolysis of nucleotide to nucleosides. It is involved in a variety of functions including the physiological control of energy balance, metabolic regulation, and cell replication [72]. By immunohistochemistry, the accumulation of cN1A in perinuclear regions and rimmed vacuoles in both sIBM and fIBM muscle fibres indicates that cN1A protein might have a potential contribution during myonuclear degradation [44]. Although exome or whole genome sequencing performed in 19 patients did not detect any protein-altering variants in both *NT5C1A* and *NT5C1B* genes [44], the genes encoding for the cN1A protein and a functionally related protein, respectively, are still important genetic candidates for understanding the relationship between sIBM autoimmunity and myofiber degeneration, and worth further genetic investigation.

### Candidate genetic factors related to degenerative changes in sIBM

#### β-amyloid (*APP*) and presenilin (*PSEN*)

It has been widely recognized that there are abnormal accumulations of beta-amyloid (Aβ) protein, N-terminal and C-terminal of beta-amyloid precursor protein (*APP*), and AβPP-mRNA in both sIBM and hIBM muscle fibres [45,73,74]. Aβ42 is considered more cytotoxic and more prone to self-association and oligomerization than Aβ40, thereby exerting a detrimental role in AD brain [75]. Similarly, increased Aβ42 oligomers were demonstrated preferentially in sIBM affected fibres [76] and also in plasma from patients with sIBM [77]. These indicate that the intra-muscle fibre accumulation of Aβ42 oligomers in sIBM contributes to the pathogenic cascade [76]. The mechanisms of overexpression of *APP* gene in sIBM have not been fully understood. The fact that mutations in exons 16 and 17 of *APP* gene have been found to be pathogenic in AD, resulting in early-onset familial AD, suggests that mutations in this gene might also play a role in sIBM. In line with a previous small study [78], no coding *APP* mutations were found in our 58 sIBM cases.

Abnormal accumulation of presenilin proteins occurs in the brains of both sporadic and familial AD patients. Autosomal dominant inheritance of mutations in presenilin 1 (*PSEN1*) and presenilin 2 (*PSEN2*) genes are also found to be responsible for early onset familial AD, with mutations in *PSEN1* gene as the most common cause [79]. The exact roles of presenilin are still not clear, but it has been considered that mutations in presenilin genes cause increased synthesis of the cytotoxic Aβ42 [80]. Abnormal accumulation of presenilin 1 protein was also reported immunohistochemically in muscle fibres of both sIBM and autosomal-recessive hIBM [46]. This indicates there might be some similarities shared between AD and IBM in pathogenic mechanism of presenilin deposits; however, mutations in *PSEN1* and *PSEN2* have not been investigated in either sIBM or hIBM cases.

#### Dysferlin (*DYSF*)

Dysferlin is a modular type II transmembrane protein newly identified as a binding partner of AβPP that co-aggregates with Aβ42 in sIBM muscle fibres [47]. In normal human muscle, dysferlin is immunohistochemically present in the muscle fibre sarcolemma and involved in plasmalemmal repair after injury as well as in T-tubule contraction and calcium homeostasis. Mutations in *DYSF* gene are known to cause a range of autosomal recessive myopathies, called dysferlinopathies including Miyoshi myopathy and limb girdle muscular dystrophy type 2B (LGMD2B) [81]. Similarly to dysferlinopathies, dysferlin is prominently reduced in the muscle fibre sarcolemma in sIBM muscle biopsies [47]. In addition, there is abnormal distribution of dysferlin into the cytoplasmic protein aggregates which co-localise with Aβ42 aggregates. Accordingly, it is suggested that mutation(s) in *DYSF* gene might link to the pathogenesis of sIBM though the possible mechanisms is unknown [47].

#### Apolipoprotein E (*APOE*)

ApoE protein deposits but not mRNA have been identified in both sIBM and hIBM muscle fibres [82,83]. The *APOE ϵ4* allele is recognized to be an important risk factor for developing late-onset AD. Several studies have been carried out to investigate the roles of *APOE* alleles in sIBM. Only one study of a cohort of 14 Australian sIBM patients suggested that *ϵ4* allele may be a susceptibility factor for the development of sIBM [84]. However, this association was not replicated in subsequent studies in Europe and USA [48,51,85-87]. A study in a larger cohort (N = 57) [48], including also a meta-analysis, argues against a role of *APOE ϵ4* allele as a susceptibility factor for developing sIBM. However, the authors demonstrated a non-significant trend towards an earlier age at onset in patients with the *APOE ϵ2* allele [48].

Our preliminary data (58 sIBM cases from UK, USA and two European countries) is consistent with the above meta-analysis with respect to the association with *APOE ϵ4* allele. Interestingly, we found a statistically significant decreased frequency of *APOE* genotype *ϵ2/ϵ3* (p-value = 0.026) in the patient group compared with our British control individuals (unpublished data), and thus we found an association between *ϵ2* allele and a reduced risk of developing sIBM (Table 4). ApoE may play a role in the AβPP pathway, as ApoE protein was found to co-localise with Congo red-positive β-amyloid deposits in sIBM cases. However, it is difficult to elucidate actual influences of *APOE* alleles on the development of sIBM, mainly because of the genetic heterogeneity of this disease.

**Table 4:** **Frequency of *APOE *** **alleles in different studies**

**Study**	**Country**	**Number of alleles (cases/controls)**	***ϵ2 *** **allele (controls)**	***ϵ3 *** **allele (controls)**	***ϵ4 *** **allele (controls)**
Garlepp et al. [84]	Australia	28/344	0.07 (0.061)	0.69 (0.811)	0.29 (0.128)
Harrington et al. [86]	UK	22/116	0 (0.086)	0.818 (0.767)	0.182 (0.147)
Love et al. [87]	UK	82/5008	0.05 (0.08)	0.79 (0.79)	0.16 (0.13)
Askanas et al. [85]	USA	22/70	0.09 (0.10)	0.86 (0.79)	0.05 (0.11)
Gossrau et al. [51]	Germany	70/112	0.1 (0.036)	0.757 (0.848)	0.143 (0.116)
Needham et al. [48]	Australia	114/344	0.044 (0.061)	0.825 (0.811)	0.132 (0.128)
Present study (our unpublished data)*	UK, USA and two European countries	116/1540	0.07 (0.13)	0.77 (0.73)	0.16 (0.14)

*All participants gave their written informed consent and the study was approved by the National Research Ethics Service (NRES) Committee London – Queen Square (REC reference: 12/LO/1557).

#### Phosphorylated Tau (*MAPT*)

Accumulation of phosphorylated-tau is widely known to be another major protein present in sIBM muscle aggregates, which is strikingly similar with those found in taupathies which share hyperphosphorylated deposited tau protein in the brain [88]. Different microtubule-associated protein tau (*MAPT*) gene mutations have been identified in many different tauopathies including AD, frontotemporal dementia and Parkinsonism linked to chromosome 17 (FTDP-17), progressive supranuclear palsy (PSP) and corticobasal degeneration (CBD). The majority of them affect exons 9–13 [89]. In addition to *MAPT* mutations, a common extended haplotype (H1) in *MAPT* appears to be a risk factor for sporadic PSP [49]. A 238 bp deletion in *MAPT* intron 9 is inherited as a part of the less common H2 sub-haplotype [49]. We analysed this deletion polymorphism (*del-In9*) in 81 sIBM DNA cases and 159 British healthy controls, but no significant differences were found between the two groups.

#### Alpha-1-antichymotrypsin (*SERPINA3*)

Alpha-1-antichymotyrpsin (α1ACT; *SERPINA3*, previously known as *ACT*) is considered a major acute-phase protein and belongs to the serpin superfamily specifically inhibiting serine proteases. Similarly to AD brains, abnormal accumulations of α1ACT, located closely with β-amyloid protein containing ubiquitinated amyloid plaques, have been described in sIBM muscle [90]. Though genotype of *SERPINA3/AA* (rs4934) was found to alter the AD risk associated with *APOE ϵ4/ϵ4* genotype [91], this correlation was not found in a cohort of 35 sIBM patients [51]. It might also be due to the small size of the patients’ cohort, which indeed lacked cases with the *APOE ϵ4/ϵ4* genotype.

#### Prion (*PRNP*)

Prion protein and mRNA (normal cellular isoform - PrPc) have been described as being abnormally accumulated in vacuolated muscle fibres in both sIBM and hIBM [92,93]. Lampe et al. reported that homozygosity for methionine at codon 129 (M/M) of the prion protein (*PRNP*) gene was more prevalent in sIBM (14 of 22 patients, 64%), when compared with controls [50]. But this finding was not replicated in later studies with larger cohorts (51.2% M/M [94] and our unpublished data 40% M/M).

#### TAR DNA-binding protein (*TARDBP*) and other heterogeneous nuclear ribonucleoproteins (hnRNPs)

A predominantly nuclear heterogeneous nuclear ribonucleoprotein (hnRNP) TAR DNA-binding protein-43 (TDP-43) is found as a major component of ubiquitinated inclusions in amyotrophic lateral sclerosis (ALS) and frontotemporal lobar dementia (FTLD-U). Mutations in *TARDBP* have been detected in both sporadic and familial forms of ALS [95]. Several recent studies [52,96-98] have detected sarcoplasmic TDP-43 inclusions in sIBM muscle fibres, which contrast with predominately nuclear immunostaining observed in normal tissue [52]. Similar TDP-43 deposits were also identified in IBMPFD, hIBM2 and other myofibrillar myopathies [97]. The similarities between these changes in sIBM muscle and those seen in brain tissue of ALS and FTLD-U suggest that sIBM may share some pathological pathways with these two diseases. TDP-43 could potentially interfere with the normal regulation of gene expression at the transcriptional level, regulating gene splicing and stabilizing extranuclear RNAs that maintain myofibre protein production. But exonic mutations in *TARDBP* gene have not yet been examined in a sIBM.

Abnormalities in the distribution of two other hnRNPs, hnRNPA1 and hnRNPA2B1, were identified in sIBM muscle fibres [53]. Similarly to TDP-43, these proteins were also depleted in myonuclei and form part of sarcoplasmic aggregates in sIBM muscle [53]. This further suggests that sIBM myofibre injury might be linked to disrupted RNA homeostasis [99], since there is depletion of these genes at the protein but not at the mRNA level [53]. Mutations in *hnRNPA1* and *hnRNPA2B1* have been identified in familial multisystem proteinopathy with similar myonuclei depletion and sarcoplasmic aggregates [100]. These genes constitute, therefore, good candidates to be further investigated in sIBM patients.

#### C9orf72 repeat

An expanded GGGGCC hexanucleotide repeat in the noncoding region of *C9orf72* has been identified as the most common genetic cause of familial ALS, FTLD or a combination of both phenotypes and TDP-43-based pathology (FTLD-TDP) [54]. In a recent study, it was suggested that mutations in *C9orf72* may involve disrupted protein degradation [101]. Therefore, we hypothesized that genetic defects in *C9orf72* might also play a role in the pathogenesis of sIBM. Fifty-eight sIBM cases were analysed for *C9orf72* repeat by our group, but no expanded repeats were found.

### Mitochondrial DNA abnormalities (mtDNA deletions) and sIBM

Mitochondrial abnormalities are another important pathological feature in sIBM muscle biopsies, consisting of ragged-red fibres and mostly showing enzyme histochemical deficiency of COX activity. These changes are more prevalent in sIBM than in polymyositis, dermatomyositis and normal ageing muscle fibres [102]. It is therefore of great interest to investigate another group of susceptibility factors – mitochondrial DNA (mtDNA). An accumulation of mtDNA molecules with large-scale deletions was found in many COX-deficient ragged-red fibres of sIBM patients (e.g. [103]), with multiple different deletions in different muscle fibres but usually one predominant type of mtDNA deletion present in each COX-deficient fibre [55,104]. Thirty-three different deletions were identified by sequencing four patients with sIBM. The majority of mtDNA deletion breakpoints identified in sIBM muscle fibres span from the region of nt8029-8032 to the region of nt16066-16078 [55] which are similar to those found in normal ageing and in autosomal dominant progressive external ophthalmoplegia (adPEO). These indicate that there may be a shared mechanism for the generation of mtDNA deletions in normal ageing, sIBM and adPEO. AdPEO and other hereditary disorders with multiple mtDNA deletions have been found associated with mutations in some nuclear genes, such as thymidine phosphorylase gene (*TYMP*, previously known as *ECGF1*), solute carrier family 25 (mitochondrial carrier; adenine nucleotide translocator) member 4 gene (*SLC25A4*, previously known as *ANT1*), chromosome 10 open reading frame 2 (*C10orf2*) and polymerase gamma 1 (*POLG1*), which are important for mtDNA maintenance and replication [16]. Putative defects in these nuclear genes may directly or indirectly affect sIBM muscle. Notwithstanding no mutations in these genes were identified in sIBM cases, there is not enough evidence to exclude these genes as possible candidates. The reason for the accumulation of mtDNA deletions in sIBM muscle fibres is still unclear and no correlation between the presence of deletions and gender, age, or the main clinical features has been found so far [16,104]. But similar to the normal ageing muscle, mtDNA mutations in sIBM may be involved in the muscle atrophy and weakness.

In addition to mtDNA deletions, mutations at mtDNA nucleotide positions 3192, 3196, 3397 and 4336, which are associated with late-onset AD, are possible risk factors for sIBM. In sIBM cases only the frequency of the common 16311C variant has been found more frequent than in AD and controls, but these differences were not statistically significant [105]. Interestingly, all the patients with 16311C variant were *HLA-DR3* positive [105], suggesting that there might be some interaction between this variant and *HLA-DR3*, which is in a genomic region strongly associated with sIBM. Further studies are required to investigate whether this variant plays a pathogenic role in sIBM and/or its possible interaction with other genetic factors.

Recently a gene called ‘Translocase of Outer Mitochondrial Membrane 40’ (*TOMM40*) which is adjacent to and in linkage disequilibrium with the *APOE* locus on chromosome 19, has been implicated in AD [106]. *TOMM40* encodes an outer mitochondrial membrane translocase facilitating the transport of unfolded proteins such as amyloid-β from the cytosol into the mitochondrial intermembrane space [107]. A polyT repeat, an intronic polymorphism (rs10524523), in the *TOMM40* gene together with the *APOE* genotypes has been shown to influence disease susceptibility of AD [108]. It has been reported that carriers of the *APOE ϵ3* allele with a very long (VL) polyT repeat alleles in *TOMM40* had reduced risk of sIBM compared to controls, and this was also associated with a later age at onset of symptoms [56]. The rs10524523 may modulate expression levels of *TOMM40* and/or *APOE* to influence disease susceptibility. This also comes to a hypothesis that genetic variants of *TOMM40* could be associated with altered mitochondrial pore function and transport of proteins into mitochondria. This could result in energy metabolism changes and increased reactive oxygen species formation, further contributing to the impairment in mitochondria and degeneration in muscle fibres [56].

## Future work – whole-exome sequencing and RNA sequencing

Over the past five years, next-generation sequencing technologies have already shown considerable promises in the identification of rare variants associated not only with Mendelian but also with complex diseases. For example, rare variants in *TREM2* were recently found to be associated with increased risk of Alzheimer’s disease by using whole-genome and whole-exome sequencing (WES) [109]. Up to recently, most genome-wide studies were based on common SNPs thought to have small effects on disease, and thus large size cohorts were needed to find positive associations. The advent of WES enables now the identification of rare coding variants which are likely functional and likely show stronger effects, increasing the likelihood of detecting disease associated variants. This advantage of WES is particularly important for rare diseases like sIBM where the number of cases is probably not large enough for a conventional genome-wide association study (GWAS). Moreover, WES also enables the analysis of the whole set of genes belonging to candidate biochemical pathways in a very quick and cost effective way. These techniques have the potential to yield very interesting results, especially to uncover previously overlooked biological processes of complex disorders, linking results from studies on molecular data, clinical phenotypes and biological disease pathways. The description of mutations in the same genes causing different diseases highlights the important role of next-generation sequencing in uncovering pleiotropic events, particularly in neurodegenerative disorders [110]. Therefore, based on the International IBM Consortium Genetic Study (IIBMCGS), WES will be performed for 200 sIBM cases and 200 normal muscle controls by our group. So far, only 19 sIBM cases have been exome sequenced in an American group [44], but no further data has been reported. Our study, currently with 160 recruited sIBM cases, is the largest sIBM cohort to date. We expected to identify a number of rare sIBM variants that cluster in disease associated genes. Replication of these findings will be verified in a further 700 sIBM cases and 2200 controls. It is also worthwhile to combine with other techniques, such as genotyping, haplotyping for *HLA* gene, fragment analysis for repeat variants, and comparative genomic hybridization (CGH) analysis for copy number variants which cannot be detected by WES.

In the future, besides genetic defects identified in sIBM, RNA sequencing in muscle tissue and lymphoblast lines will identify transcriptomic profiles and expression quantitative trait loci (eQTL) in sIBM and other inflammatory myopathies. The application of RNA sequencing will also allow us to study and dissect splicing abnormalities which may not be detected by exon arrays. Investigating the transcriptomic and genomic factors will be important in revealing dysregulated molecular pathways in these disorders, identifying a diagnostic signature in blood and revealing biomarkers for future therapeutic strategies. A searchable database to the research community is also planned to build up to integrate WES, RNA sequencing and eQTL data, further to aligned with the biobank database with disease progression, response to treatment and muscle imaging.

## Conclusion

In conclusion, sIBM remains an enigmatic muscle disorder even though it has been about half a century since the first case was described. The primary pathogenesis of sIBM is still controversial, but it is more likely to due to multifactorial mechanisms, including immune changes, degenerative changes, mitochondrial abnormalities and the ageing milieu in the muscle fibres, which involve a complex interaction between environmental triggers (possibly viral) and genetic susceptibility. Despite the strikingly phenotypic similarities between sIBM muscle fibres and the brains of AD, research on the candidate genes encoding the deposited proteins have not shown any positive result so far. Genes located within the MHC region are still the most strongly associated candidates with sIBM. Genes linked to hIBM and pathologically sIBM-like myopathies may provide important clues for sIBM genetic research. Effective WES and RNA sequencing analysis of a large sIBM cohort will significantly contribute to our knowledge in this under-explored area.

### Search strategy

References for this Review were identified by searches in PubMed between 1969 and May 2014. The search terms “inclusion body myositis”, “inclusion body myopathy”, “IBM”, “rimmed vacuolar myopathy”, and “inflammatory myopathy” were used. References were also identified from the bibliography of the identified articles. Only papers published in English or with available English translations of relevant data were reviewed. The final reference list was generated on the basis of relevance to the topics covered in this Review.

## Competing interests

The authors declare that they have no competing interests.

## Authors’ contributions

QG contributed to the literature search and to drafting the manuscript. All authors contributed to the critical revision process and approved the final manuscript. MGH, HH, and PM are principal investigators of the International IBM Consortium Genetic Study (IIBMCGS).
